# Current state of multiparameter magnetic resonance imaging of the prostate

**DOI:** 10.1590/S1679-45082018MD4408

**Published:** 2018-06-22

**Authors:** Thais Caldara Mussi

**Affiliations:** 1 Hospital Israelita Albert Einstein, São Paulo, SP, Brazil.

**Keywords:** Prostate/diagnostic imaging, Magnetic resonance imaging, Próstata/diagnóstico por imagem, Ressonância magnética

## Abstract

Magnetic resonance imaging of the prostate is an imaging method that has shown increasing relevance in urological practice. Due to technological evolution of scanners and the introduction of functional sequences, it has enabled greater accuracy in detection and characterization of prostate tumors.

## RESUMO

A ressonância magnética de próstata é um método de imagem que tem demonstrado crescente relevância na prática urológica. Devido aos avanços tecnológicos dos equipamentos e à introdução de sequências funcionais, possibilita maior acurácia na detecção e caracterização de tumores prostáticos.

## INTRODUCTION

**Figure 1:**
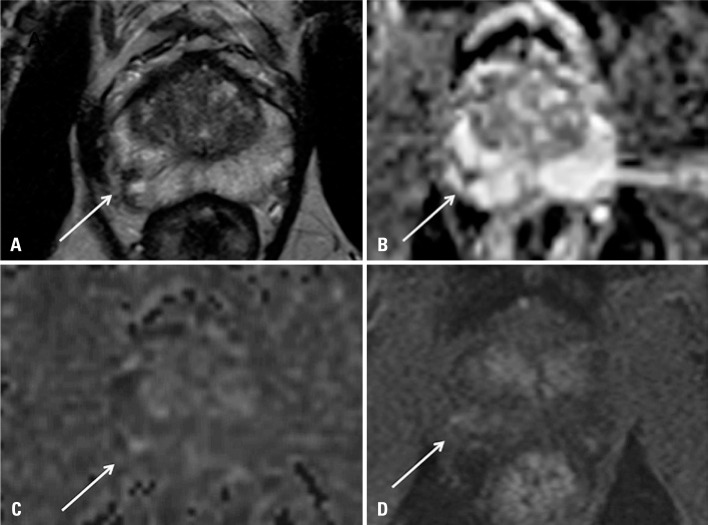
Contrast axial magnetic resonance imaging of the prostate (A) T2-weighted; (B) apparent diffusion coefficient (ADC) map; (C) diffusion sequence and (D) perfusion sequence, showing a small nodule suspected to be a clinically significant malignancy in the periphery of the right lobe (arrows).

Magnetic resonance imaging (MRI) of the prostate is an imaging method that has shown increasing relevance in urological practice since the beginning of its clinical use. Initially, prostate MRI was used for locoregional staging of patients with known malignancy.^(^ ^1^ ^)^ However, technological advances in scanners and the introduction of functional sequences have enabled greater accuracy in detection and characterization of clinically significant prostate tumors (Figure 1), with an estimated sensitivity of 74% and specificity of 88% in the literature, as demonstrated in a meta-analysis.^(^ ^2^ ^)^

Since the establishment of MRI as a method with high tumor detection rates, new technologies have emerged with the purpose of using this information to improve diagnosis of prostate cancer. Magnetic resonance imaging-guided prostate biopsy is the technique with the greatest applicability.

There are basically three methods to conduct an MRI-guided biopsy. The first is known as in-bore, *i.*e., inside the MRI scanner. The second is cognitive ultrasound (US)-MRI fusion, in which the radiologist reviews MRI scans before performing the US and collects a sample from the probable topography of the lesion during conventional transrectal biopsy. The third is true US-MRI fusion: a hardware coupled to the US equipment performs real-time, automated merging of US images with previously acquired MRI scans. This modality currently has the best cost/effectiveness ratio for this purpose.^(^ ^3^ ^)^ Some studies have already demonstrated the superiority of MRI-guided US biopsy for diagnosing clinically significant prostate neoplasms when compared to random conventional transrectal biopsy.^(^ ^4^ ^)^

To support the increasing application of the method in the management of these patients, a consensus between the Society of Abdominal Radiology (SAR) and the American Urological Association (AUA) was published in 2016, suggesting that patients with a negative prostate biopsy but persisting clinical suspicion must be submitted to MRI if a high-quality scan is available and a new biopsy is under consideration.^(^ ^5^ ^)^

In addition, MRI is suggested for patients meeting clinical, laboratory and histological criteria for an active surveillance protocol (low-risk tumors), as well as for their follow-up. Magnetic resonance imaging has negative predictive values above 90% to rule out clinically significant malignancy, and reclassification rates that reach 60% when the MRI is positive. However, the definition of disease progression and the cost-effectiveness of the method are not yet fully established.^(^ ^6^ ^)^

In 2012, the European Society of Urogenital Radiology (ESUR) developed guidelines to standardize the acquisition, interpretation and reporting of prostate MRI scans, the Prostate Imaging Reporting and Data System, better known by its acronym: PI-RADS.^(^ ^7^ ^)^ In 2015, these criteria were jointly revised by the American College of Radiology (ACR) and the AdMeTech Foundation, leading to the development of the original PI-RADS proposal (PI-RADS version 2).^(^ ^8^ ^)^ A recent study has demonstrated positivity values for clinically significant tumors using the PI-RADS version 2 of 15.7%, 33.0%, 70.5% and 90.7% for PI-RADS categories 2, 3, 4 and 5, respectively.^(^ ^9^ ^)^

In sum, the benefits of prostate MRI in increasing detection of clinically significant tumors are already established prior to a new biopsy, before the patient is included in an active surveillance protocol (and during follow-up), and even before a first biopsy. However, the method needs to be better studied, with prospective randomized trials, to be established as a screening tool. Nowadays, in order to improve the applicability and economic viability of MRI, the literature suggests that the scan be performed without an endorectal coil, in 1.5 Tesla scanners and with no injection of contrast medium.^(^ ^10^ ^)^

The advances of MRI aim to add value in care of patients with prostate cancer, granting this method a central role in the clinical management of this prevalent disease.
